# Institutional Notes and News

**Published:** 1911-09-09

**Authors:** 


### INSTITUTIONAL NOTES AND NEWS.

**Insurance Bill and Leicester Saturday Society.**

At the half-yearly meeting of the Leicester and County Saturday Hospital Society, over which Sir Edward Wood presided, the report showed that there was no abatement of interest, and that 1,400 workpeople had been added to the list of systematic contributors during the six months. The Society's convalescent home at Desford had received 319 male members, and 297 male and female members had been sent to other homes. Sir Edward Wood stated that the new home for female members at Woodhouse Eaves was in a state of advancement, and that he was proud of the achievements of the Society. As Chairman of the Leicester Infirmary he felt the great indebtedness the institution owed for the increasing help it received from the workpeople. There had been considerable perturbation among the hospitals as to the effect of the National Insurance Bill. Personally he had the strongest possible faith in the organisation of the Saturday Hospital Society, and while he was prepared to find a small decrease in the contributions of the workpeople until the new Act, if passed, had developed into the smooth waters of satisfactory administration, he had the utmost confidence that after the first few months the Society would be in an equally good position—and possibly better than at present. The report was supported by Mr. S. A. Gimson and Mr. J. Gipson Clarke (vice-presidents), Dr. Pratt, and several delegates, and adopted.

**The Position at Worthing Hospital.**

The coming opening of a new out-patient department for the Worthing Hospital lent unusual interest to the annual meeting of the Governors which was held at the Town Hall. Mr. W. H. B. Fletcher, J.P., one of the vice-presidents (in the absence of the Duke of Norfolk, the President), took the chair. Mr. H. R. P. Wyatt moved the adoption of the annual report and statement of accounts, which showed a deficit of £267. As regards the new out-patients' department, which has become an absolute necessity, about £1,060 has been received or promised, and as the contract for the building was £2,760, another £1,700 is still required. It is hoped that the Duke of Norfolk will attend the opening ceremony, but it is regretfully stated that the Duchess will be unable to accompany him. The opening will probably take place in November, but the work has been delayed owing to the strike and the consequent difficulty of getting material.

Mr. Wyatt then made a strong appeal for more subscriptions, and said that he thought many of the tradespeople might do more, and that there were also scores of people living in big houses in the town whose names one looked for in vain on the subscription list. It would be interesting to know whether this was due to callousness or whether anybody disapproved of the hospital, and, if so, for what reason. Mr. Wyatt also stated that the new out-patients' department would increase the annual expenditure, and that unless more support was forthcoming they would find themselves in a very serious financial position. Mr. Simpson also made an emphatic appeal for funds. The report was adopted, and the Committee of Management re-elected, as was also Capt. Montague Prentice, the secretary, who, since his comparatively recent appointment, has been busily engaged in securing funds for the new out-patients' quarters. The architect is Mr. A. Morris Butler, A.R.I.B.A., of Worthing.

